# A robust internal control for high-precision DNA methylation analyses by droplet digital PCR

**DOI:** 10.1186/s13148-018-0456-5

**Published:** 2018-02-21

**Authors:** Heidi D. Pharo, Kim Andresen, Kaja C. G. Berg, Ragnhild A. Lothe, Marine Jeanmougin, Guro E. Lind

**Affiliations:** 1 Department of Molecular Oncology, Institute for Cancer Research, Oslo University Hospital, the Norwegian Radium Hospital, PO Box 4950, Nydalen, NO-0424 Oslo, Norway; 2 KG Jebsen Colorectal Cancer Research Centre, Oslo University Hospital, Oslo, Norway; 3 Centre for Cancer Biomedicine, Faculty of Medicine, University of Oslo, Oslo, Norway; 4 Department of Biosciences, The Faculty of Mathematics and Natural Sciences, University of Oslo, Oslo, Norway

**Keywords:** Digital PCR, Internal control, Methylation, Normalization, PoDCall, 4Plex

## Abstract

**Background:**

Droplet digital PCR (ddPCR) allows absolute quantification of nucleic acids and has potential for improved non-invasive detection of DNA methylation. For increased precision of the methylation analysis, we aimed to develop a robust internal control for use in methylation-specific ddPCR.

**Methods:**

Two control design approaches were tested: (a) targeting a genomic region shared across members of a gene family and (b) combining multiple assays targeting different pericentromeric loci on different chromosomes. Through analyses of 34 colorectal cancer cell lines, the performance of the control assay candidates was optimized and evaluated, both individually and in various combinations, using the QX200™ droplet digital PCR platform (Bio-Rad). The best-performing control was tested in combination with assays targeting methylated *CDO1*, *SEPT9*, and *VIM*.

**Results:**

A 4Plex panel consisting of *EPHA3*, *KBTBD4*, *PLEKHF1*, and *SYT10* was identified as the best-performing control. The use of the 4Plex for normalization reduced the variability in methylation values, corrected for differences in template amount, and diminished the effect of chromosomal aberrations. Positive Droplet Calling (PoDCall), an R-based algorithm for standardized threshold determination, was developed, ensuring consistency of the ddPCR results.

**Conclusion:**

Implementation of a robust internal control, i.e., the 4Plex, and an algorithm for automated threshold determination, PoDCall, in methylation-specific ddPCR increase the precision of DNA methylation analysis.

**Electronic supplementary material:**

The online version of this article (10.1186/s13148-018-0456-5) contains supplementary material, which is available to authorized users.

## Background

Digital PCR (dPCR) enables absolute quantification of nucleic acids. The principle behind the method was described already in 1992 [1], but its use was for many years hampered by lack of suitable protocols and instrumentation. Technology development during the last decade has led to several commercial systems for dPCR, resulting in a rapid increase in the publication rate of dPCR studies [2, 3]. With the concomitant increase in liquid biopsy analyses for cancer screening, for detection of minimal residual disease after surgery, and for monitoring cancer patients, the need for high-precision analyses of circulating tumor-derived nucleic acid molecules is obvious, but not necessarily implemented.

With the dPCR technology, a PCR mixture can be randomly divided into a large number of partitions. Individual PCRs are performed inside each partition, and based on the fraction of fluorescence-positive partitions, the absolute quantity of the target can be calculated [2]. One of the most commonly used platforms is the droplet digital PCR (ddPCR), where the partitions are represented by thousands of nanoliter-scale droplets, formed by water-in-oil emulsion [4–6].

The sample partitioning inherent for ddPCR considerably reduces the competition from any background DNA, allowing detection of minimal amounts of a target of interest. The sensitivity is in principle only limited by the number of droplets analyzed [4], and the method has been demonstrated to trace one mutated gene copy in the background of 200,000 wild-type molecules [7]. This makes ddPCR particularly valuable for analyses of various types of non-invasive biomarkers, such as detection of *KRAS* mutations in the blood of colorectal cancer patients, predicting lack of response to targeted treatment [8], screening for metastatic breast cancer by small increases in *HER2* copy number in plasma samples [9], gene expression analyses to detect hepatocellular carcinoma from circulating tumor cells [10], and detection of bladder cancer among hematuria patients [11].

Although the ddPCR technology has great potential also for DNA methylation analyses, only few studies have been published so far [12–16]. The lack of consensus regarding how to perform standardized experiments might be a contributing factor. Generation of consistent methylation data is dependent on the use of a suitable control for normalization, as previously demonstrated for other PCR-based DNA methylation analyses [17, 18]. The aims of the present study were to develop a robust internal control for ddPCR DNA methylation analyses and demonstrate its value in terms of increased precision of the normalized methylation data.

## Methods

### DNA from cancer cell lines

DNA from 34 colorectal cancer cell lines (Caco2, CL-11, CL-34, CL-40, Co115, Colo205, Colo320, Colo678, DLD-1, EB, FRI, HCC2998, HCT116, HCT15, HT29, IS1, IS3, KM12, LoVo, LS1034, LS174T, NCI-H508, RKO, SW1116, SW1463, SW403, SW48, SW480, SW620, SW837, SW948, TC71, V9P, and WiDr) was isolated using either a standard phenol-chloroform protocol or a magnetic bead approach (Maxwell® 16 System; Promega). Cell lines were either purchased from cell line repositories or kindly provided by collaborators, as previously described [19]. Authentication of the cell lines was performed by short tandem repeat testing, as reported by Ahmed et al. [20]. DNA concentrations were measured using a NanoDrop 1000 Spectrophotometer (Thermo Fisher Scientific). DNA copy number data (Affymetrix Genome-Wide Human SNP 6.0 microarrays) were available for all cell lines [19].

### Bisulfite conversion

The EpiTect Bisulfite Kit (Qiagen) was used for bisulfite conversion of 1.3 μg DNA according to the manufacturers’ standard protocol. After conversion in the MJ Mini Personal Thermal Cycler (Bio-Rad Laboratories), the samples were automatically purified and eluted in 40 μl elution buffer by the QIAcube System (Qiagen).

### Design and development of candidate internal controls

With the aim of developing a control for methylation-specific ddPCR that targeted multiple non-CpG containing loci located on different chromosomes, two approaches were tested. In the first approach, “A,” a common sequence shared by several members of a gene family (the Aldolase A family; *ALDOA*, and the Cytochrom C family; *CYCS*) was targeted. This approach implied introduction of only one control assay into the target gene reaction, with the rationale of reducing the chances of interference with target amplification. In the second approach, “B,” multiple assays, targeting different loci in the exonic part of various genes located close to the centromeres (*n* = 13; *ALDH1B1*, *ANKRD30A*, *EPHA3*, *HAO2*, *IGFBPL1*, *ITGAD*, *KBTBD4*, *MRPS5*, *NIPA2*, *PLEKHF1*, *SAMSN1*, *SYT10*, and *TTC5*), were designed and tested in different combinations. These control gene candidates were chosen from a list of pericentromeric reference genes, previously suggested by Bio-Rad to be stable in regard to copy number variations [21]. This approach implied introduction of several control assays into the target gene reaction (see Additional file 1: Table S1 for assay sequences and their chromosomal locations). The best-performing control (VIC-labeled) was tested in combination with assays targeting methylated *CDO1*, *SEPT9*, and *VIM* (FAM-labeled), through ddPCR analyses of 34 colorectal cancer cell lines. Finally, the performance of the control was compared to two previously published single locus controls, *ACTB* [22] and C-LESS [23].

### Digital droplet PCR

The QX200™ Droplet Digital™ PCR System (Bio-Rad) was used for analyses. The ddPCR reaction mixture consisted of 1x ddPCR Supermix for Probes (Bio-Rad), 900 nM of each primer, 250 nM of the probe, and approximately 30 ng bisulfite-converted DNA template, in a final volume of 22 μl. Droplets were generated by the QX200 Droplet Generator (Bio-Rad), using 20 μl of the ddPCR mixture and 70 μl droplet generation oil (Bio-Rad). Samples were transferred to a 96-well PCR plate (Bio-Rad) and sealed in the PX1 PCR Plate Sealer (Bio-Rad). The PCR was performed in a T100 Thermal Cycler (Bio-Rad; see Additional file 1: Table S2 for PCR cycling conditions). The fluorescence signals were measured by the QX200 Droplet Reader (Bio-Rad). For each experiment, the following control samples were included: two methylation-positive controls (commercially available in vitro methylated DNA (IVD); Zymo Research), one methylation-negative control (bisulfite-treated DNA from normal blood of healthy donors), one non-bisulfite-converted IVD sample, and a non-template control (NTC; water). All analyses were performed according to the digital MIQE guidelines (Additional file 2) [24].

### Data analyses

**Fig. 1:**
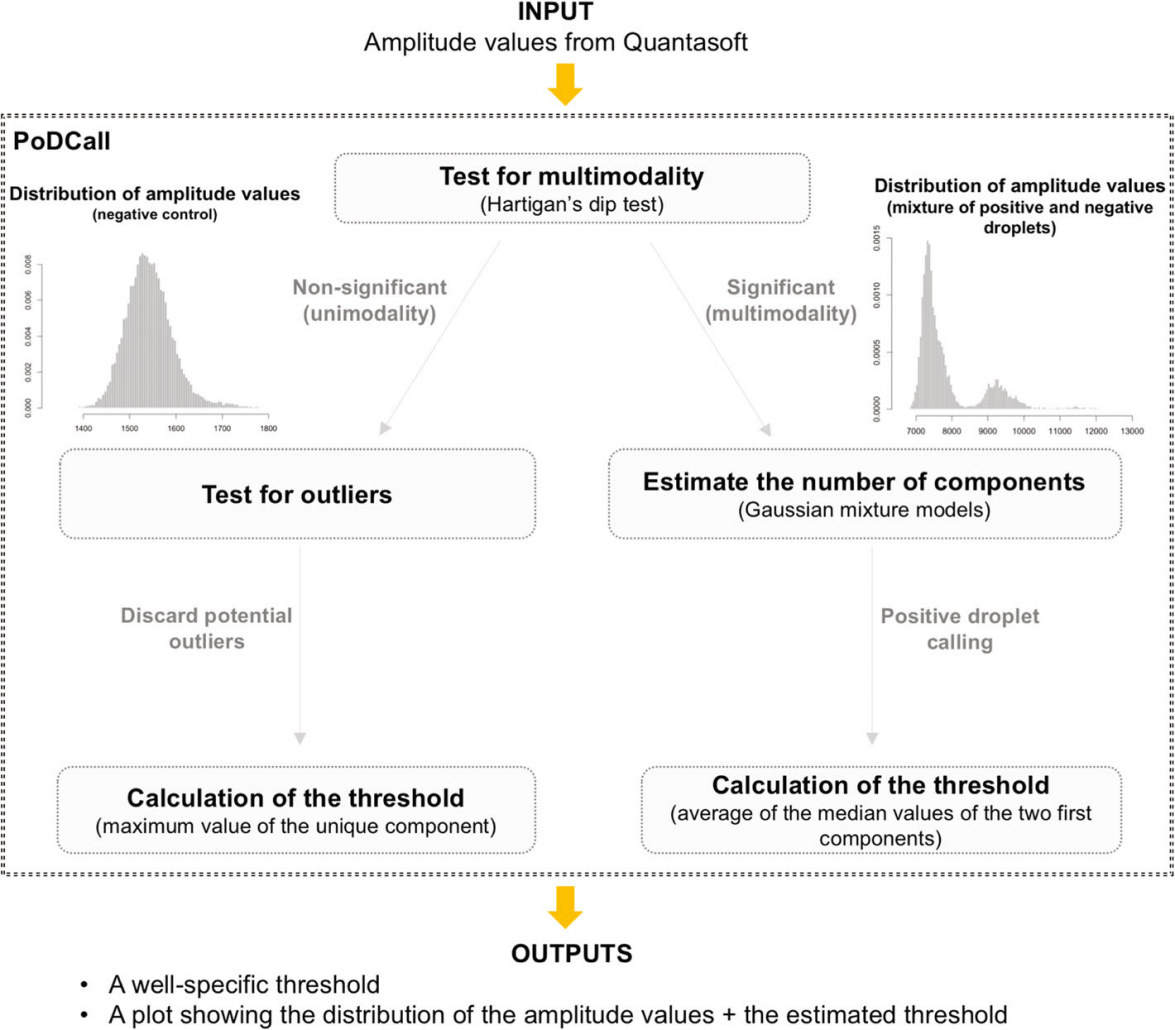
Overview of PoDCall workflow. The flowchart displays the main steps of the PoDCall algorithm in order to determine the threshold for calling positive droplets for individual wells

Data from the QX200 Droplet Reader was analyzed in QuantaSoft version 1.7.4.0917 (Bio-Rad). The Positive Droplet Calling (PoDCall) algorithm was developed in-house to determine well-specific thresholds that discriminated positive droplets, i.e., containing the target, from negative droplets, i.e., did not contain the target. The PoDCall workflow is illustrated in Fig. 1 and summarized in the following. Amplitude values were extracted from QuantaSoft and used as input data for PoDCall. First, the multimodality of the distribution of the amplitude values was assessed using Hartigan’s dip test [25]. Two strategies were applied depending on the significance of the test. If non-significant, i.e., *p* value > 0.05, the distribution was assumed to be unimodal and the threshold was set as the maximum amplitude value, after testing for potential outliers. Alternatively, if the test was significant, a Gaussian mixture model was fitted to the distribution of droplets, using the R package “mclust,” version 5.2.3 [26]. Next, the number of mixture components in the distribution was assessed by a likelihood ratio test, whose significance was approximated by using a bootstrap approach with 700 replications. Finally, the threshold was defined as the average value between the modes of the first and second components. The resulting thresholds, representing the output data from PoDCall, were manually entered in QuantaSoft. Based on the fraction of positive droplets, concentrations of methylated copies/μl were calculated by the software. Normalized concentrations were generated by dividing the concentration of the target gene on the concentration of the control. These normalized values were then multiplied by a constant, i.e., the mean concentration of the control among all analyzed cell lines, in order to have them in the same range as the non-normalized concentration.

### Statistics

The statistical analyses were performed using R version 3.2.2. In order to investigate how normalized concentrations were affected by chromosomal aberrations, cell lines were stratified according to the presence of deletions, gains, deletions and gains (both), or no aberration. Differences in mean among the groups were investigated using ANOVA.

## Results

### The 4Plex panel is the best-performing control

The gene family approach for designing an internal control (approach A) provided poor results (Additional file 1: Figure S1) and was discarded from further analyses. The *ALDOA* assay resulted in an IVD concentration of ~ 400 copies/microliter (Additional file 1: Figure S1A), which was lower than expected given the number of loci targeted by this assay (*n* = 3; Additional file 1: Table S1). For *CYCS*, no positive droplet band was detected (Additional file 1: Figure S1B).

**Fig. 2:**
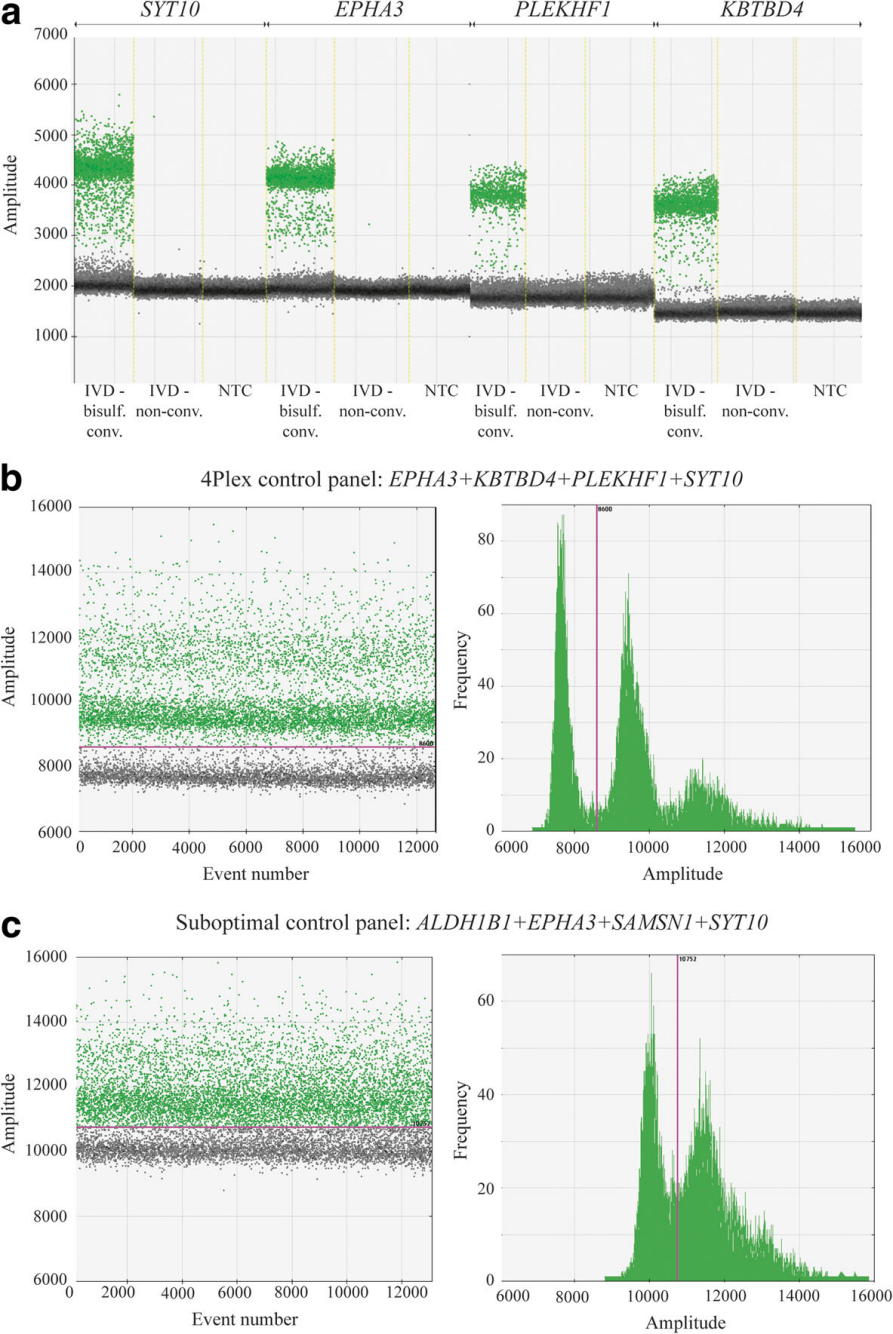
Droplet dPCR amplification plots for individual and combined control assays. **a** Individual amplification patterns for *SYT10*, *EPHA3*, *PLEKHF1*, and *KBTBD4* comprising the 4Plex. Positive droplets are shown in green and negative in black/gray. **b** Fluorescence amplitude plots for the 4Plex in a representative cell line. The pink line represents the threshold, dichotomizing positive and negative droplets. Left plot: the amplitude value (*y*-axis) for individual droplets (*x*-axis) is depictured (positive droplets, green; negative droplets, black/gray). Right plot: the frequency of droplets (*y*-axis) at each fluorescence amplitude value (*x*-axis) is shown. Positive droplets have an amplitude value to the right of the threshold and negative droplets, amplitude value to the left of the threshold. **c** Fluorescence amplitude plots for an alternative control panel consisting of *ALDH1B1*, *EPHA3*, *SAMSN1*, and *SYT10*. Abbreviations: IVD, in vitro methylated DNA; NTC, non-template control (i.e., water)

For the approach that combined single assays targeting different loci in the exonic part of various pericentromeric genes (approach B), nine (*ALDH1B1*, *EPHA3*, *IGFBPL1*, *KBTBD4*, *MRPS5*, *PLEKHF1*, *SAMSN1*, *SYT10*, and *TTC5*) of the 13 designed assays showed a clear separation between positive and negative droplets (Fig. 2a and Additional file 1: Figure S2). *EPHA3*, *KBTBD4*, *PLEKHF1*, and *SYT10* had similar amplitude value of the negative droplet cluster (around 2000; Fig. 2a), and merging these assays into a control panel resulted in clear separation between positive and negative droplets (Fig. 2b). Combinations with other assays, e.g., *ALDH1B1* and *SAMSN1*, which had a higher amplitude value of the negative droplet cluster (~ 2500–2800; Additional file 1: Figure S2), resulted in reduced separation (Fig. 2c). Thus, the four-assay panel consisting of *EPHA3*, *KBTBD4*, *PLEKHF1*, and *SYT10*, termed the 4Plex, was identified as the best-performing control. Across all samples analyzed, the 4Plex provided a consistent amplification pattern, with V9P as an exception. This cell line displayed a shift in the droplet pattern, comprising a significant reduction of the negative droplet peak, and simultaneous increase of the positive droplet peak (Additional file 1: Figure S3).

### The 4Plex has minor impact on amplification of the target gene

**Fig. 3:**
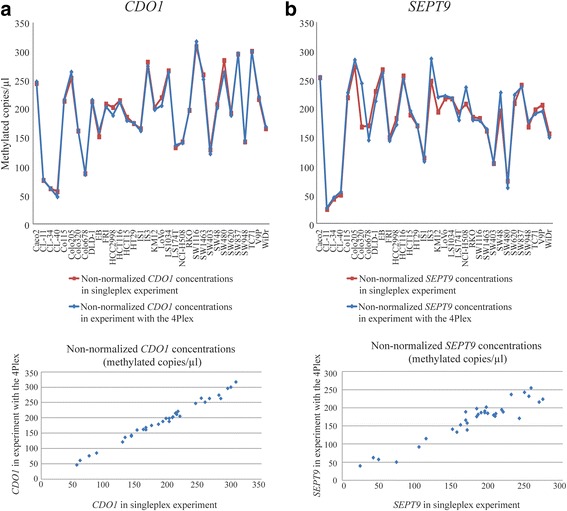
The 4Plex has minor effects on the non-normalized concentrations of the target. **a** Non-normalized *CDO1* concentrations from a singleplex experiment, and from an experiment with the 4Plex, are plotted. Upper plot: *x*-axis: the 34 colorectal cancer cell lines. *y*-axis: non-normalized *CDO1* concentrations in methylated copies/microliter. Results from the singleplex experiment are shown in red and from the experiment with the 4Plex in blue. Lower panel: *x*-axis: non-normalized *CDO1* concentrations from a singleplex experiment. *y*-axis: non-normalized *CDO1* concentrations from an experiment with the 4Plex. Each dot represents one cell line. **b** The same plots as in **a**, but for *SEPT9*

The assays comprised in the 4Plex are labeled with VIC and run in the same reaction as the FAM-labeled assays that measure the methylation of *CDO1*, *SEPT9*, and *VIM*. To evaluate whether the presence of the 4Plex had an impact on the amplification of the target gene, non-normalized target gene concentrations (methylated copies/μl) from experiments with and without the 4Plex control were compared. The resulting non-normalized concentrations were highly consistent for both *CDO1* and *SEPT9* (Fig. 3). For *VIM*, discrepancies between the concentrations resulting from the experiments with and without the 4Plex were observed (median absolute difference of 21%). However, this was comparable with resulting discrepancies from using the alternative controls *ACTB* [22] (median absolute difference of 23%) and C-LESS [23] (median absolute difference of 38%; Additional file 1: Figure S4).

### The 4Plex corrects for differences in template amounts and can act as a template-loading control

**Fig. 4:**
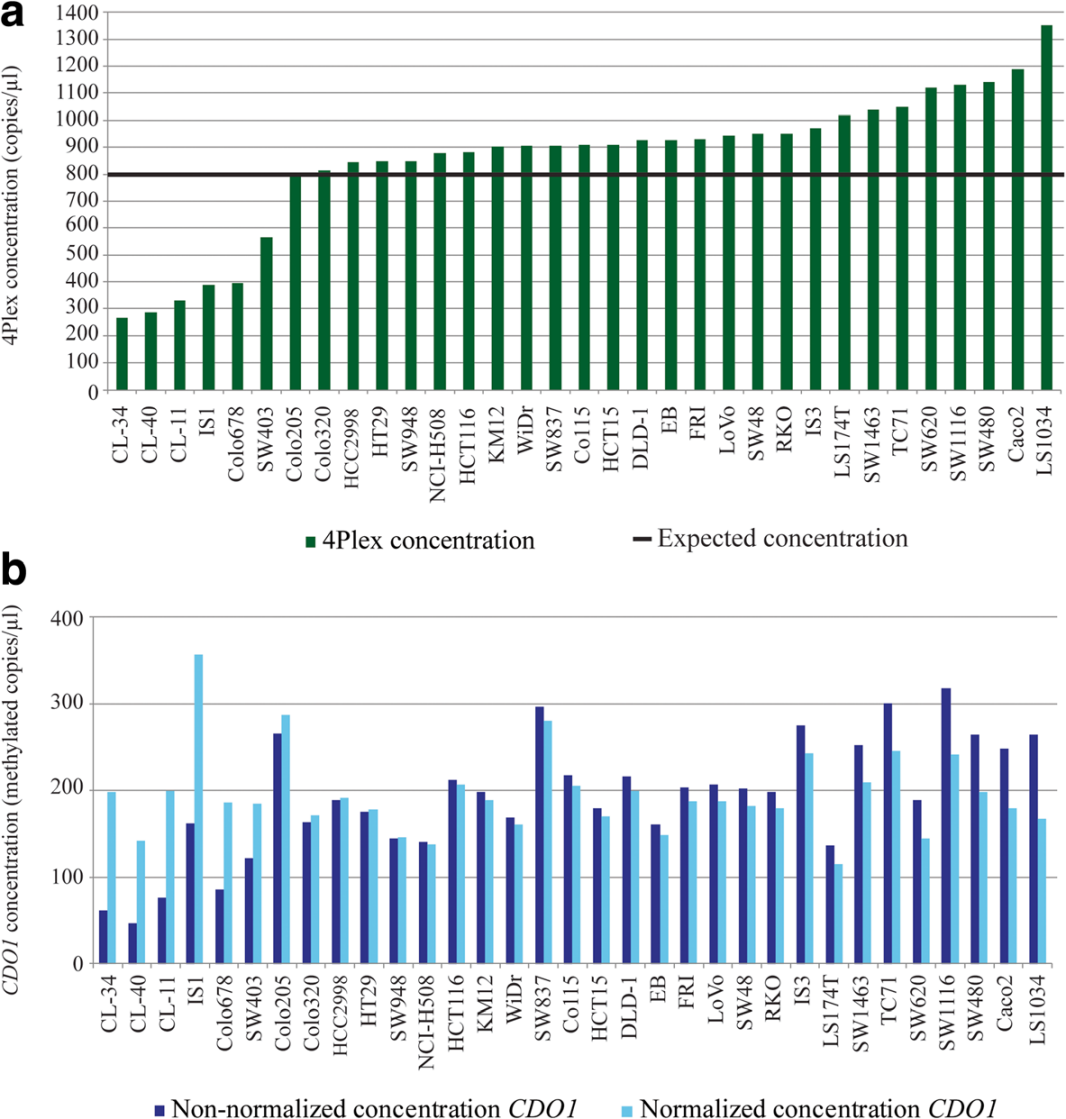
The 4Plex corrects for variable template amount. **a** The 4Plex concentrations across the cell line panel. The horizontal black line at 800 copies/μl indicates the expected 4Plex concentration. **b** Non-normalized (dark blue) and normalized (light blue) *CDO1* concentrations (methylated copies/μl) are shown for the same cell lines as in **a**

**Fig. 5:**
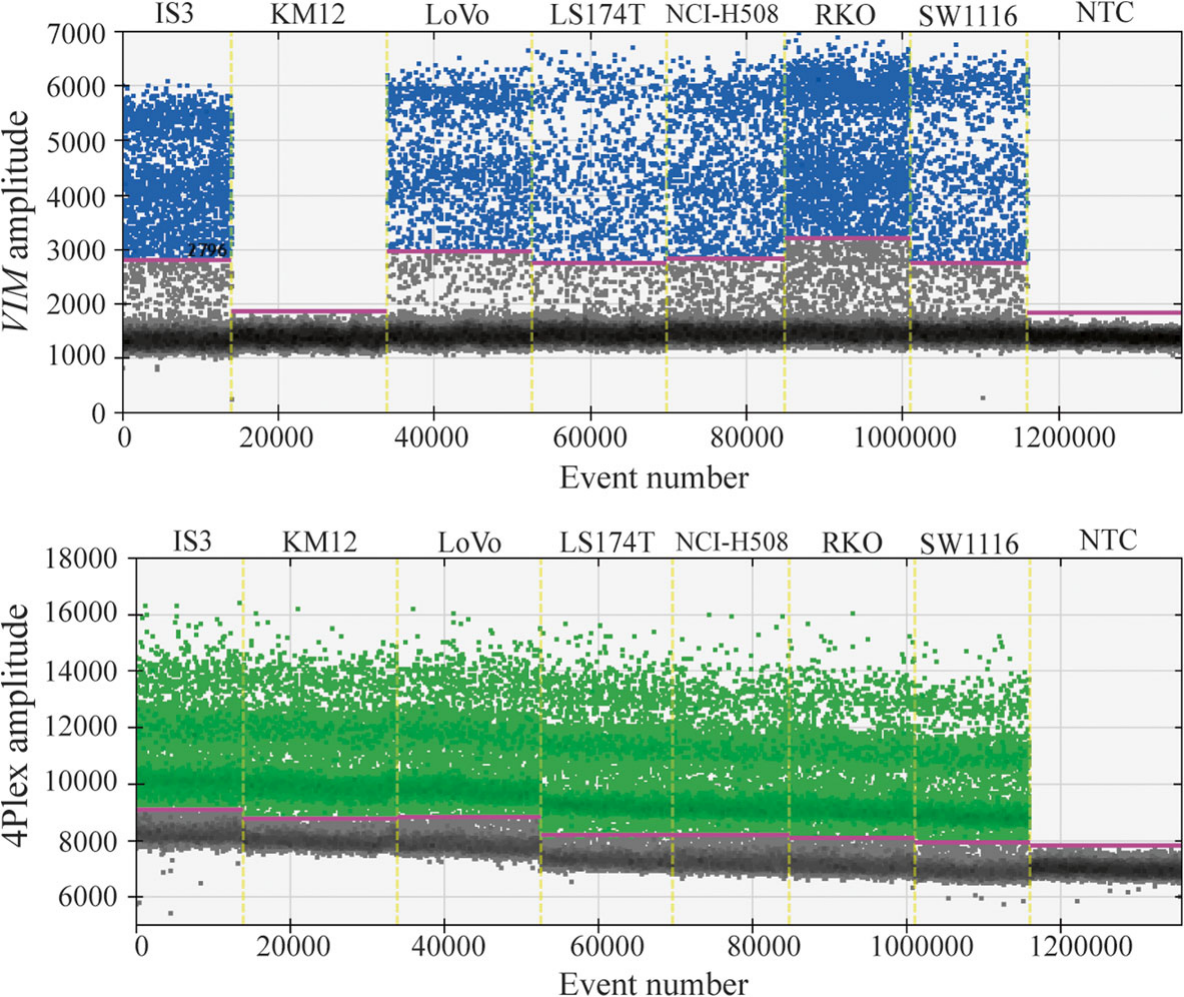
The 4Plex acts as a template-loading control that prevents erroneous scoring of template-negative samples. Upper panel: amplification of *VIM* in a selection of cell lines (blue, positive droplets; black/gray, negative droplets). Lower panel: amplification of the 4Plex in the same cell lines (green, positive droplets; black/gray, negative droplets). Water is used as a non-template control (NTC)

Since the target assays in the present study (*CDO1*, *SEPT9*, and *VIM*) are designed to amplify methylated sequences only, the 4Plex represents an internal control for normalization for these analyses. Despite the use of the same theoretical input amount for all samples in this work (based on the input in the bisulfite conversion), the 4Plex revealed concentration differences across the cell line panel (Fig. 4a). Moreover, comparing non-normalized and 4Plex-normalized concentrations of the target genes across the cell line panel, large differences were observed for the samples with the highest and lowest 4Plex concentrations (Fig. 4b). Finally, inclusion of the 4Plex discriminates true methylation-negative samples (e.g., KM12; Fig. 5) from potential false methylation-negative samples lacking template (NTC; Fig. 5).

### 4Plex-normalized concentrations show less variance than non-normalized target gene concentrations

**Fig. 6:**
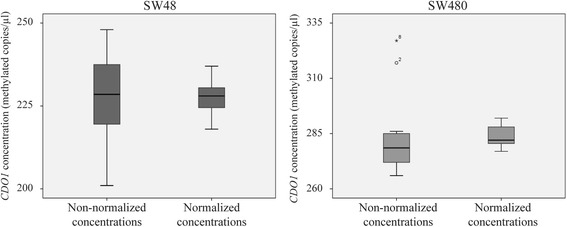
The 4Plex-normalized concentrations show lower variance than non-normalized concentrations. Results are shown for *CDO1* in two different cell lines (SW48 and SW480). Each analysis includes 12 replicates

Non-normalized and 4Plex-normalized concentrations of the target genes were compared among replicates of two different samples (SW48 and SW480). For both samples, normalized concentrations of *CDO1* showed lower variance than the non-normalized concentrations (Fig. 6; 28.5 vs. 183 for SW48 and 20.3 vs. 356 for SW480). The same tendency of reduced variability after normalization was seen for *SEPT9* and *VIM* (Additional file 1: Figure S5).

### Normalization by the 4Plex diminishes the effect of chromosomal aberrations

**Fig. 7:**
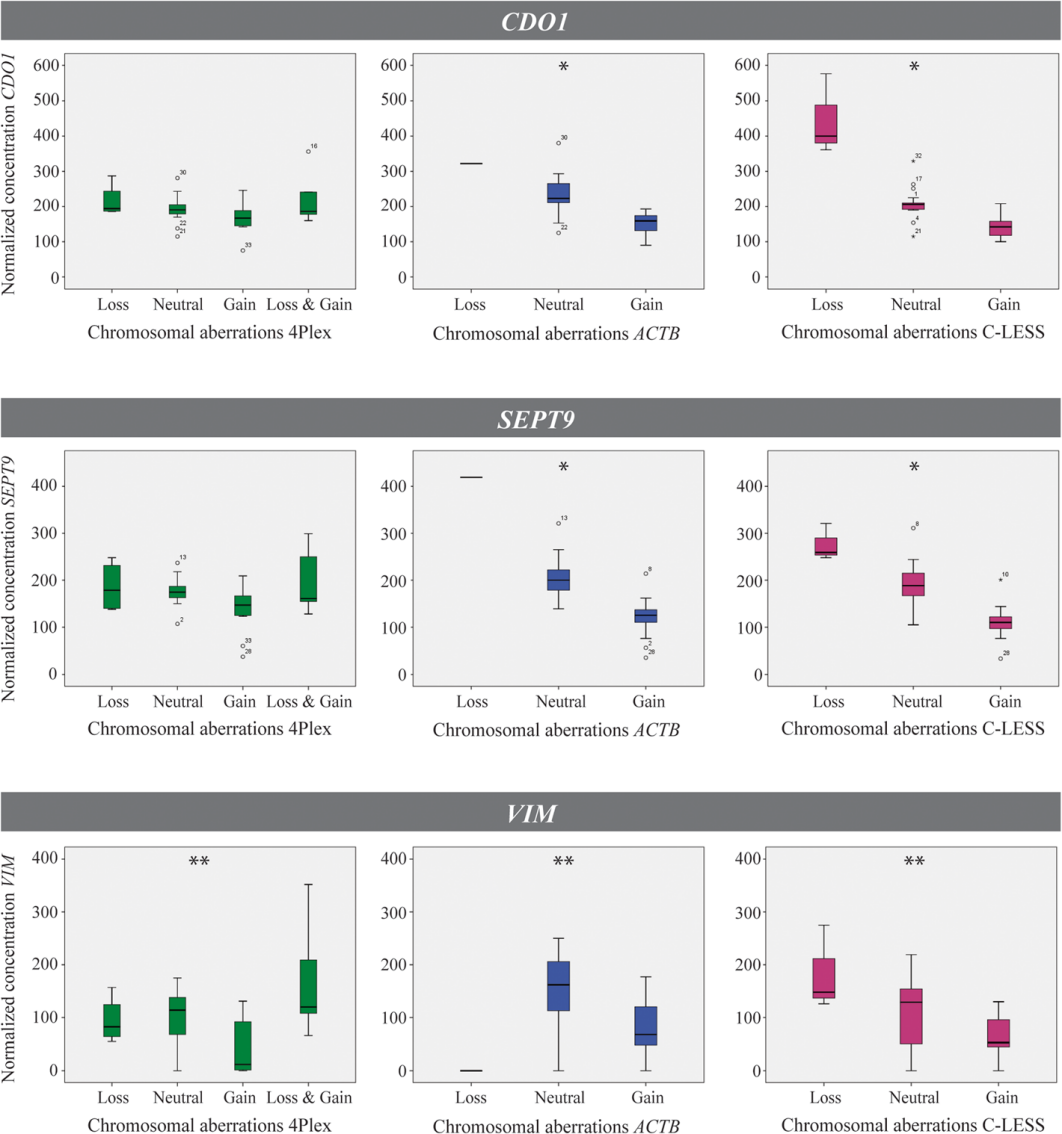
Normalization by the 4Plex reduces the effect of chromosomal aberrations. Normalized concentrations of *CDO1* (upper panel), *SEPT9* (middle panel), and *VIM* (lower panel) are shown for three different internal controls: 4Plex (green), *ACTB* (blue), and C-LESS (pink). Cell lines are grouped after their chromosomal aberration status of the respective controls (*x*-axis). *Significant at a 1% level. **Significant at a 5% level

To evaluate the potential impact of chromosomal aberrations on the 4Plex compared to the previously suggested single locus controls *ACTB* [22] and C-LESS [23], the normalized concentrations of the target genes were compared in groups of colorectal cancer cell lines harboring no aberrations, gain, loss, or both gain and loss in the control loci (Additional file 1: Table S3). As shown in Fig. 7, chromosomal aberrations significantly affected the *ACTB*-normalized concentrations (blue boxes) of *CDO1*, *SEPT9*, and *VIM* (*P* < 0.001, *P* < 0.001, and *P* = 0.016, respectively) as well as the C-LESS-normalized concentrations of the same target genes (pink boxes; *P* < 0.001, *P* < 0.001, and *P* = 0.012, respectively). In contrast, the 4Plex (green boxes) was found to diminish the effect of chromosomal aberrations when analyzing these three target genes (*P* = 0.131, *P* = 0.109, and *P* = 0.011).

## Discussion

The ddPCR technology allows highly sensitive quantification of nucleic acids and has great potential for analyses of DNA methylation. In the present work, we have developed a robust internal control for methylation-specific ddPCR, the 4Plex. This control consists of four individual pericentromeric assays and is analyzed in the same reaction as the target of interest. We demonstrate that normalization using the 4Plex standardizes the results by increasing the precision of the target quantification. Such precision is especially important for the rapidly evolving field of liquid biopsies, which holds great promise for disease detection, monitoring, and emergence of drug resistance [27].

Two different strategies are typically used for robust quantification of methylated targets in ddPCR analyses. In line with standard mutation/SNP assays, primers binding to bisulfite converted DNA, independent of the methylation status, can be paired with probes with different fluorescent marks, one binding to the methylated version and the other to the unmethylated version of the target sequence. With such a design, the ratio between methylated and unmethylated DNA can be determined, and the use of an internal control would be limited to normalizing for minor technical variations, such as pipetting inaccuracies. This represents a convenient design for absolute quantification, but can be challenging for DNA methylation analyses where the number of CpGs in the target region of interest, e.g., gene promoters, is often high, and the presence of such CpG sites in the primer binding sites may bias the amplification. A commonly used alternative, often seen in qMSP/MethyLight analyses, is designing an assay where both primers and probe bind exclusively to the methylated version of the target. This type of analysis requires an internal control for normalization, preferably reflecting the total amount of amplifiable template in the reaction.

In the present study, two approaches for developing such an internal control for normalization in ddPCR analyses were evaluated. The gene family approach (A) provided poor results for both alternatives tested. Amplification was either failing or resulting in a lower than expected concentration (Additional file 1: Figure S1). The latter could potentially be explained by a degenerated base in the sense primer, causing less efficient binding to one or more of the targeted loci. In contrast, approach (B), consisting of combining several individual assays into a combined control panel, was successful and resulted in the 4Plex.

Using the 4Plex as an internal control in methylation-specific ddPCR has several advantages. In addition to reducing the overall variability in methylation values and increasing the reproducibility, the 4Plex can adjust for unforeseen variations in the experimental pipeline. Although equal amounts of DNA, as measured by the NanoDrop, were loaded into the bisulfite treatment and subsequent ddPCR reaction in the present study, the 4Plex revealed significant DNA concentration differences across samples (Fig. 4a). Normalization by the 4Plex thereby prevented over- and underestimation of methylation levels (Fig. 4b). This is highly relevant for analyses of clinical material, where the DNA quality and integrity is typically varying [28].

As expected, and in line with single locus references, the 4Plex served as a template-loading control that allowed distinguishing between true methylation-negative samples and template-negative samples (Fig. 5). Moreover, as the 4Plex consists of four assays and has a considerably higher concentration than the target gene, it could also be used to establish a lower threshold for allowing scoring of samples. With a very low signal from the control, it is unlikely that the reaction contains enough template to detect potential methylation. Such a lower loading threshold, revealing samples that cannot be robustly determined, will reduce the number of false negatives.

Chromosomal aberrations are common in various diseases, and cancer in particular [29, 30], and will affect the normalization if present in the control locus [17, 18]. The importance of using an internal control that targets multiple loci is known from qMSP/MethyLight analyses [17, 18] and was recently also emphasized by Uehiro and colleagues for ddPCR DNA methylation analyses [13]. In MethyLight, the transposable ALU element, containing more than one million copies spread out in the human genome, represents a robust internal control. However, for ddPCR methylation analyses, the ALU element is too abundant and saturates the reaction (data not shown). The 4Plex on the other hand amplifies four loci in the genome, located on different chromosomes, without reaching saturation. When used as an internal control in the present study, the 4Plex reduced the effect of chromosomal aberrations on normalized methylation values of the target gene. In contrast, the use of the single locus controls *ACTB* and C-LESS caused significant deviations in methylation values (Fig. 7). An additional advantage with the 4Plex is that it only quantifies template that can be amplified by the targeted assays, i.e., bisulfite-converted DNA, in contrast to the C-LESS control that amplifies its target independent of bisulfite conversion status [23].

To evaluate whether the presence of the 4Plex had an impact on the amplification of the target gene, the target was run alone and in combination with the 4Plex. The resulting methylation concentrations were highly consistent for two of three genes tested (*CDO1* and *SEPT9*; Fig. 3). Interestingly, for *VIM*, the discrepancies from using the 4Plex were smaller than the observed discrepancies from using the single locus controls *ACTB* and C-LESS (Additional file 1: Figure S4).

In ddPCR methylation analyses, rain, i.e., droplets that fall between the positive and negative clusters, is a known phenomenon [12, 13, 31] (visible in Figs. 2 and 5), making methylation concentrations sensitive to inconsistent threshold determination. To standardize the analyses, PoDCall, an algorithm for automated threshold calculations, was developed (Fig. 1). PoDCall contributed to standardization through well-specific scoring of positive and negative droplets and thus increased the consistency of the methylation data. However, it is likely that PoDCall will be applicable also for other types of ddPCR analyses where rain is observed. Finally, PoDCall was also useful in order to correct for unexpected technical artifacts in the ddPCR analyses, such as shifts in baseline fluorescence between samples (Additional file 1: Figure S6). Improved accuracy of ddPCR analyses by using automated threshold determination has also been underscored by others [32, 33] but has to our knowledge received limited recognition.

The 4Plex performed well across all samples analyzed, with V9P as an exception (Additional file 1: Figure S3). This is most likely explained by a significant chromosomal amplification observed for the *PLEKHF1* locus in this cell line. In contrast, a “normal” droplet distribution pattern was seen across a series of more than 100 colorectal cancer tissues (data not shown), indicating that such pattern aberrations are rare. Furthermore, the 4Plex was successfully used in recent analyses of non- to minimally invasive material from bladder cancer- and cholangiocarcinoma patients, respectively (Pharo et al., unpublished, Vedeld et al., unpublished), underscoring that this control can be applied across cancer types.

## Conclusions

In conclusion, the 4Plex internal control increases the precision of methylation-specific ddPCR analyses by reducing the variability in methylation concentrations, correcting for variable input amount, and by reducing the effect of chromosomal aberrations. PoDCall, the algorithm for automated threshold determination, contributes to additional consistency of the ddPCR results. We advocate for implementation of the 4Plex and PoDCall as a standard in methylation-specific ddPCR analyses.

## Additional files

Additional file 1: **Table S1.** Sequence information for the ddPCR assays used in the present study. **Table S2.** The PCR thermal cycling conditions (T100 Thermal Cycler, Bio-Rad). **Table S3.** Gene copy number states of *ACTB*, C-LESS, and the 4Plex in the 34 colorectal cancer cell lines. **Figure S1.** Droplet dPCR amplification of a non-CpG containing sequence shared by members of a gene family (approach A) provides poor results. **Figure S2.** Individual control assay candidates from approach B. **Figure S3.** The 4Plex shows a consistent amplification pattern across the cell line panel with V9P as an exception. **Figure S4.** Non-normalized *VIM* concentrations are lower with a control assay included in the reaction. **Figure S5.** A tendency of lower variation in 4Plex-normalized target gene concentrations is seen in replicates of the same sample. **Figure S6.** PoDCall, the algorithm for automated threshold determination, corrects for shifts in baseline fluorescence between samples and performs better than the QuantaSoft software. (DOCX 791 kb)

Additional file 2: dMIQE checklist for authors, reviewers, and editors. (DOCX 262 kb)

## Abbreviations

ddPCR: Droplet digital PCR
DNA: Deoxyribonucleic acid
dPCR: Digital PCR
IVD: In vitro methylated DNA
NTC: Non-template control
PCR: Polymerase chain reaction
qMSP: Quantitative methylation-specific PCR
