# Supplementary material for: A robust internal control for high-precision DNA methylation analyses by droplet digital PCR
Source: Clin Epigenetics. 2018 Feb 21;10:24. doi: 10.1186/s13148-018-0456-5 (PMC5822558; doi:10.1186/s13148-018-0456-5)
Supplement: Supplementary file 1 — Table S1. Sequence information for the ddPCR assays used in the present study. Table S2. The PCR thermal cycling conditions (T100 Thermal Cycler, Bio-Rad). Table S3. Gene copy number states of ACTB, C-LESS, and the 4Plex in the 34 colorectal cancer cell lines. Figure S1. Droplet dPCR amplification of a non-CpG containing sequence shared by members of a gene family (approach A) provides poor results. Figure S2. Individual control assay candidates from approach B. Figure S3. The 4Plex shows a consistent amplification pattern across the cell line panel with V9P as an exception. Figure S4. Non-normalized VIM concentrations are lower with a control assay included in the reaction. Figure S5. A tendency of lower variation in 4Plex-normalized target gene concentrations is seen in replicates of the same sample. Figure S6. PoDCall, the algorithm for automated threshold determination, corrects for shifts in baseline fluorescence between samples and performs better than the QuantaSoft software. (DOCX 791 kb) [file 13148_2018_456_MOESM1_ESM.docx]

Additional File 1

**Additional Tables**

| **Gene** | **Sequence accession number** | **Chromosome location** | **Amplicon length** | **Sense primer** | **Antisense primer** | **Probe** | **Reference** |
| --- | --- | --- | --- | --- | --- | --- | --- |
| Control candidate assays – Approach A; locus shared by members of a gene family | | | | | | | |
| *ALDOA* | NM_000034 | 3, 10, 16 | 171 | GGA TTA TWT TTT TGT TTG GAG GTT AGA | CAA CCT TCA AAT TCT CCT TCT TCC | 6VIC-GTT TTT GTT TTG AAG GTT TG-MGB | * |
| *CYCS* | NM_018947 | 6, 7, 8 | 152 | ATG GGT GAT GTT GAG AAA GGT AAG A | ACT ATA TAA AAA TAT CCA TTT TCC TAA CCT ATC TTC | 6VIC-AGT ATA AGA TTG GGT TAA ATT TTT ATG G-MGB | * |
| Control candidate assays – Approach B; combine single loci on different chromosomes | | | | | | | |
| *ALDH1B1* | NM_000692 | 9 | 108 | GAG ATT TTG GAT AAT GGG AAG TTT TTT | ACC ATA CCA CTT ATC AAC CCA ACC | 6VIC-TTT TGG ATT TGG ATG AGG TTA-MGB | * |
| *ANKRD30A* | NM_052997 | 10 | 101 | GGA ATA TTT GAT GAG GTT GTA TTT TTG G | CTT TCC ACC AAA AAT ACA ACC TCA | 6VIC-TTT GAT ATA GTT GAA AGT TTG GTG G-MGB | * |
| *EPHA3* | NM_005233 | 3 | 99 | GGA TTT ATT AGG TGT GTA ATG TTA TGG ATT | ACT CCA CAT AAA TCT TCT AAA CTA AAT TCC T | 6VIC-TTG GTT GAG AAT AAA TTG GGT TT-MGB | * |
| *HAO2* | NM_016527 | 1 | 118 | GGT ATT TGA GAG ATG TGT TTG AGG TG | TCC CCA TCA AAC CAA ACA AAA C | 6VIC-ATA ATT TAA GGG GAG GAG ATT A-MGB | * |
| *IGFBPL1* | NM_001007563 | 9 | 96 | GAA GTT TTT TGA GGG TAT TTA AGT ATT GGA | ACC TCA TAA TCA AAA AAA CCC CCT | 6VIC-AGT TGT TTG GGG ATT ATG TT-MGB | * |
| *ITGAD* | NM_005353 | 16 | 122 | TTT AGG GAT TTA TTG TTA GGT AGG AGT TG | CTT CTC CTA CAA CTA CTT CTA AAT ACT ACC AAA | 6VIC-TGT TTA AGG TGG ATA ATT TTG T-MGB | * |
| *KBTBD4* | NM_001318724 | 19 | 86 | TTT GTA TGT GGT GGG AGG GTT T | ACA AAA AAA CAC ACC ACT CCC AA | 6VIC-TAT GTG GAA GTG TAA TAA TG-MGB | * |
| *MRPS5* | NM_001321995 | 2 | 78 | TGG GAT AGG GAG TAG GTT ATG TTA GGT | AAA ACC CAC CCA CAC CAA AC | 6VIC-TGG TGA AAG TTT TTA GTT TGT G-MGB | * |
| *NIPA2* | NM_001008860 | 15 | 106 | GTT TTT AGT TTT TTG TGT GAA GGG TTT | AAA ACT CAA CAA CAA AAT CCA AAC C | 6VIC-TTA TTA AGG AGT TGT TTG TAG GGA A-MGB | * |
| *PLEKHF1* | NM_024310 | 11 | 100 | GTA GTT TTA GAT GGT TTT TTG AGT TGG A | CAC TCC CAT CCT ATC TTC CCT CTA TA | 6VIC-AGG GAT TAG AGT AGG TTT G-MGB | * |
| *SAMSN1* | NM_022136 | 21 | 122 | GAT GAG GAA GAT GGA GAG AAT GTT TAT T | CTC TAT CCA CTA TAA AAA CTA TCC ATA AAA TCA C | 6VIC-AGT GAT TTT GTG ATT GGG A-MGB | * |
| *SYT10* | NM_198992 | 12 | 94 | GAG GTA AAT GTA GGT TTT TAG TGT TGA TTT T | CTT TAT CCT CCC AAT ACT AAT TAT TAT TTC TCC | 6VIC-AGT ATG GGT ATA GAA TTT GT-MGB | * |
| *TTC5* | NM_138376 | 14 | 82 | GAG ATA TAT AGT GTT GAG GAT GTT GG GAG | CTT CTT CCA TCT ACT ATA AAA TTT TCT CCA TC | 6VIC-AAG TAA TAG GAT GTG TAG AAG GA-MGB | * |
| Previously suggested control assays | | | | | | | |
| *ACTB* | Y00474 | 7 | 133 | TGG TGA TGG AGG AGG TTT AGT AAG T | AAC CAA TAA AAC CTA CTC CTC CCT TAA | 6VIC-ACC ACC ACC CAA CAC ACA ATA ACA AAC ACA-MGB | (1) |
| C-LESS-C1 | ** | 20 | 68 | TTG TAT GTA TGT GAG TGT GGG AGA GA | TTT CTT CCA CCC CTT CTC TTC C | 6VIC -CTC CCC CTC TAA CTC TAT-MGB | (2) |
| Target genes | | | | | | | |
| *CDO1* | NM_001801 | 5 | 101 | CGA ATT ATA GCG GCG GAG GT | AAA TCG CGT AAA CTC CGC G | 6FAM-CGT TAG GTC GGG CGG T-MGB | (3) |
| *SEPT9* | NM_001113493 | 17 | 98 | CGC GCG ATT CGT TGT TTA TTA | CCA ACC CAA CAC CCA CCT T | 6FAM-GGA TTT CGC GGT TAA C-MGB | (4) |
| *VIM* | NM_003380 | 10 | 106 | GGT CGA GTT TTA GTC GGA GTT ACG T | CCC GAA AAC GAA ACG TAA AAA CTA | 6FAM-CGT ATT TAT AGT TTG GGT AGC GC-MGB | (4) |

**Table S1:** **Sequence information for the ddPCR assays used in the present study.** All sequences are listed in 5’ to 3’ direction. Primers were purchased from BioNordika Bergman, and probes from Life Technologies. *Assay is designed for the present study. **Obtained from the NCBI Build 36.2; chromosome 20, 19199387–19199455 (5).

Reference List to Additional Table S1

1. Costa, V. L. *et al.* TCF21 and PCDH17 methylation: An innovative panel of biomarkers for a simultaneous detection of urological cancers. *Epigenetics.* **6**, 1120-1130 (2011).
2. Weisenberger, D. J. *et al.* DNA methylation analysis by digital bisulfite genomic sequencing and digital MethyLight. *Nucleic Acids Res.* **36**, 4689-4698 (2008).
3. Andresen, K. *et al.* Novel target genes and a valid biomarker panel identified for cholangiocarcinoma. *Epigenetics.* **7**, 1249-1257 (2012).
4. Ahmed, D. *et al.* A tissue-based comparative effectiveness analysis of biomarkers for early detection of colorectal tumors. *Clin.Transl.Gastroenterol.* **3**, e27 (2012).
5. Campan, M. *et al.* MethyLight. *Methods Mol Biol* . **507**, 325-37 (2009).

| C**ycling step** | **Temperature, °C** | **Time** | **Ramp rate** | **Number of cycles** |
| --- | --- | --- | --- | --- |
| Enzyme activation | 95 | 10 min | 2°C/sec | 1 |
| Denaturation | 94 | 30 sec |  | 40 |
| Annealing/extension | 60 | 1 min |  | 40 |
| Enzyme deactivation | 98 | 10 min |  | 1 |
| Hold | 4 | Infinite |  | 1 |

**Table S2: The PCR thermal cycling conditions (T100 Thermal Cycler, BioRad).** The conditions are recommended from the manufacturer.

|  | **Genomic loci** | | | | | |
| --- | --- | --- | --- | --- | --- | --- |
| **Cell lines** | ***ACTB*** | **C-LESS** | ***EPHA3*** | ***SYT10*** | ***KBTBD4*** | ***PLEKHF1*** |
| Caco2 | 0 | 0 | 0 | 1 | 0 | 0 |
| CL-11 | 1 | 0 | 0 | 0 | 0 | 0 |
| CL-34 | 1 | 0 | 0 | 0 | 0 | 0 |
| CL-40 | 0 | 0 | 0 | 1 | 0 | 0 |
| Co115 | 0 | 0 | 0 | 0 | 0 | 0 |
| Colo205 | 0 | 1 | -1 | 0 | 0 | 0 |
| Colo320 | -1 | 0 | 0 | 1 | 0 | 0 |
| Colo678 | 1 | 0 | 0/-1* | 1 | 0 | 0 |
| DLD-1 | 0 | 0 | -1 | 0 | 0 | 0 |
| EB | 0 | 1 | 0 | 1 | 1 | 0 |
| FRI | 1 | -1 | 0 | 0 | 0 | -1 |
| HCC2998 | 1 | 0 | 0 | 0 | 0 | 0 |
| HCT116 | 0 | 0 | 0 | 0 | 0 | 0 |
| HCT15 | 0 | 0 | 0 | 0 | 0 | 0 |
| HT29 | 0 | 0 | -1 | 1 | 1 | 1 |
| IS1 | 1 | 1 | -1 | 1 | 0 | -1 |
| IS3 | 1 | 0 | 0 | 0 | 0 | 0 |
| KM12 | 0 | 0 | 0 | 0 | 0 | 0 |
| LoVo | 1 | 0 | 0 | 0 | 0 | 0 |
| LS1034 | 0 | 1 | 0 | 1 | 1 | 0 |
| LS174T | 1 | 0 | 0 | 0 | 0 | 0 |
| NCI-H508 | 0 | 1 | 0 | 0 | 0 | 0 |
| RKO | 0 | 1 | 0 | 0 | 0 | 0 |
| SW1116 | 1 | -1 | -1 | 0 | 1 | -1 |
| SW1463 | 0 | 1 | 0 | 1 | 0 | 0 |
| SW403 | 1 | 1 | 0/-1* | 0 | 0 | -1 |
| SW48 | 1 | 0 | 0 | 0 | 0 | 0 |
| SW480 | 1 | 1 | 0 | 1 | 0 | 1 |
| SW620 | 1 | 1 | 0 | 1 | 1 | 0 |
| SW837 | 0 | -1 | 0 | 0 | 0 | 0 |
| SW948 | 1 | 1 | 0 | 0 | 1 | 0 |
| TC71 | 0 | 0 | 0 | 1 | 0 | 0 |
| VP9 | 1 | 1 | 0 | 0 | 0 | 1 |
| WiDr | 0 | 1 | -1 | 1 | 1 | 1 |

**Table S3: Gene copy number states of *ACTB*, C-LESS and the 4Plex in the 34 colorectal cancer cell lines.** DNA copy number data from Affymetrix SNP6.0 arrays were preprocessed by the PennCNV protocol (adapted to Affymetrix arrays) and segmented using the R copynumber package. Gene copy number estimates of <-0.15 were called as loss, and >0.15 as gain, here given as gain = 1, neutral = 0 and loss = -1. *Cell lines indicated to have two different copy number states in one gene displayed a breakpoint in the gene, thereby the resulting different copy number state.

**Additional Figures**


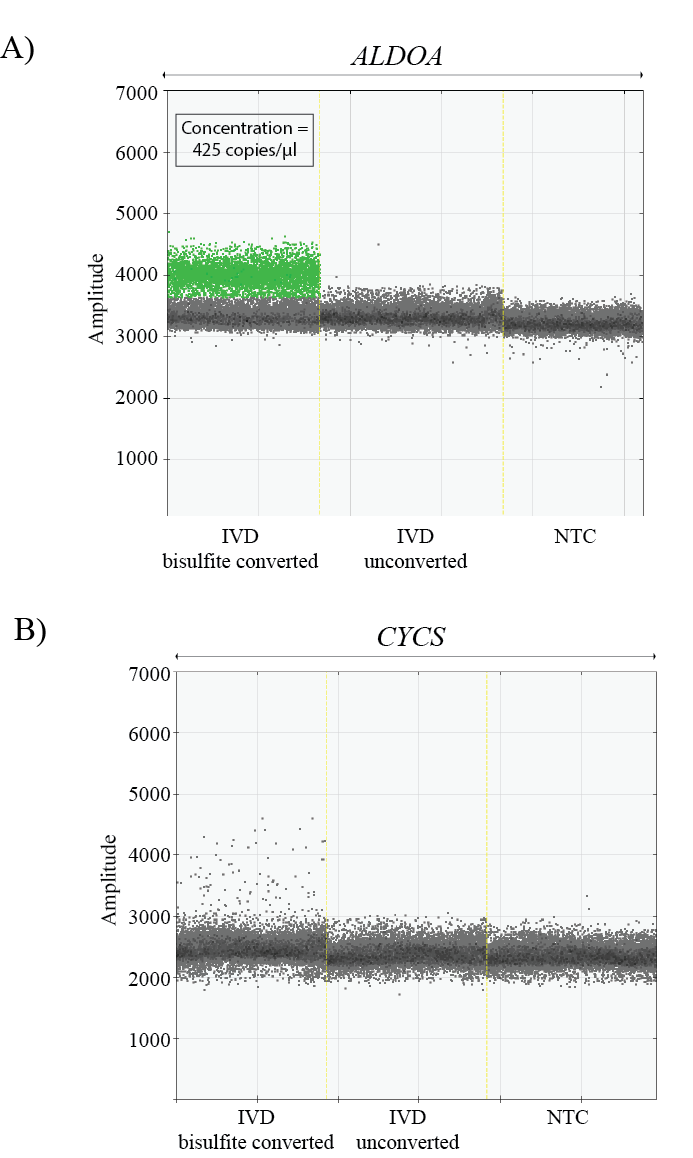


**Figure S1:** **Droplet dPCR** **amplification of a non-CpG containing sequence shared by members of a gene family (approach A) provides poor results. (A)** Amplification of the *ALDOA* (Aldolase A family) assay results in a concentration of 425 copies/µl, which is lower than expected based on the input amount and the number of targeted loci in the genome. **(B)** Amplification of the *CYCS* (Cytochrome C family) assay results in lack of a positive droplet band.


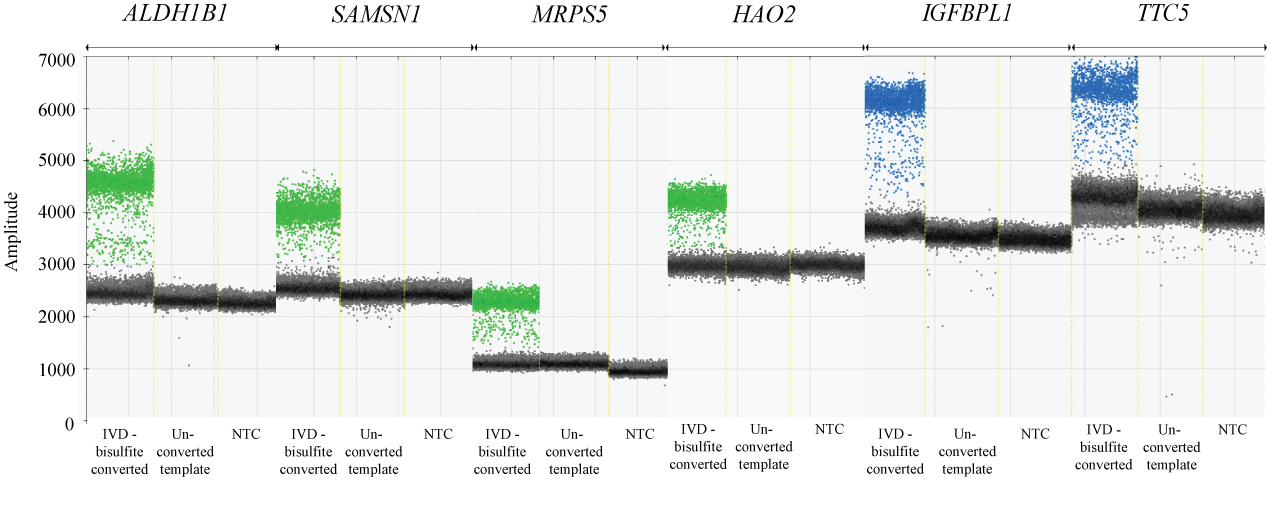


**Figure S2: Individual control assay candidates from approach B.** Positive droplets are shown in green and blue, and negative in black/grey.

**
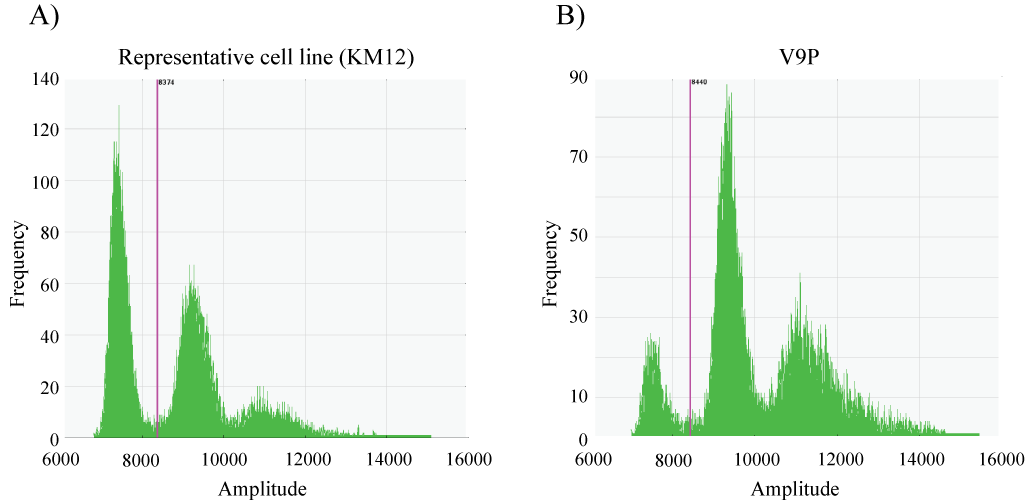
**

**Figure S3: The 4Plex shows a consistent amplification pattern across the cell line panel with V9P as an exception.** X-axis: the fluorescence amplitude value of the 4Plex. Y-axis: the frequency of droplets at each fluorescence value. The pink vertical line represents the threshold, dichotomizing droplets into negative (amplitude value left of the threshold) and positive (amplitude value right of the threshold). **(A)** A representative cell line (KM12) **(B)** V9P.


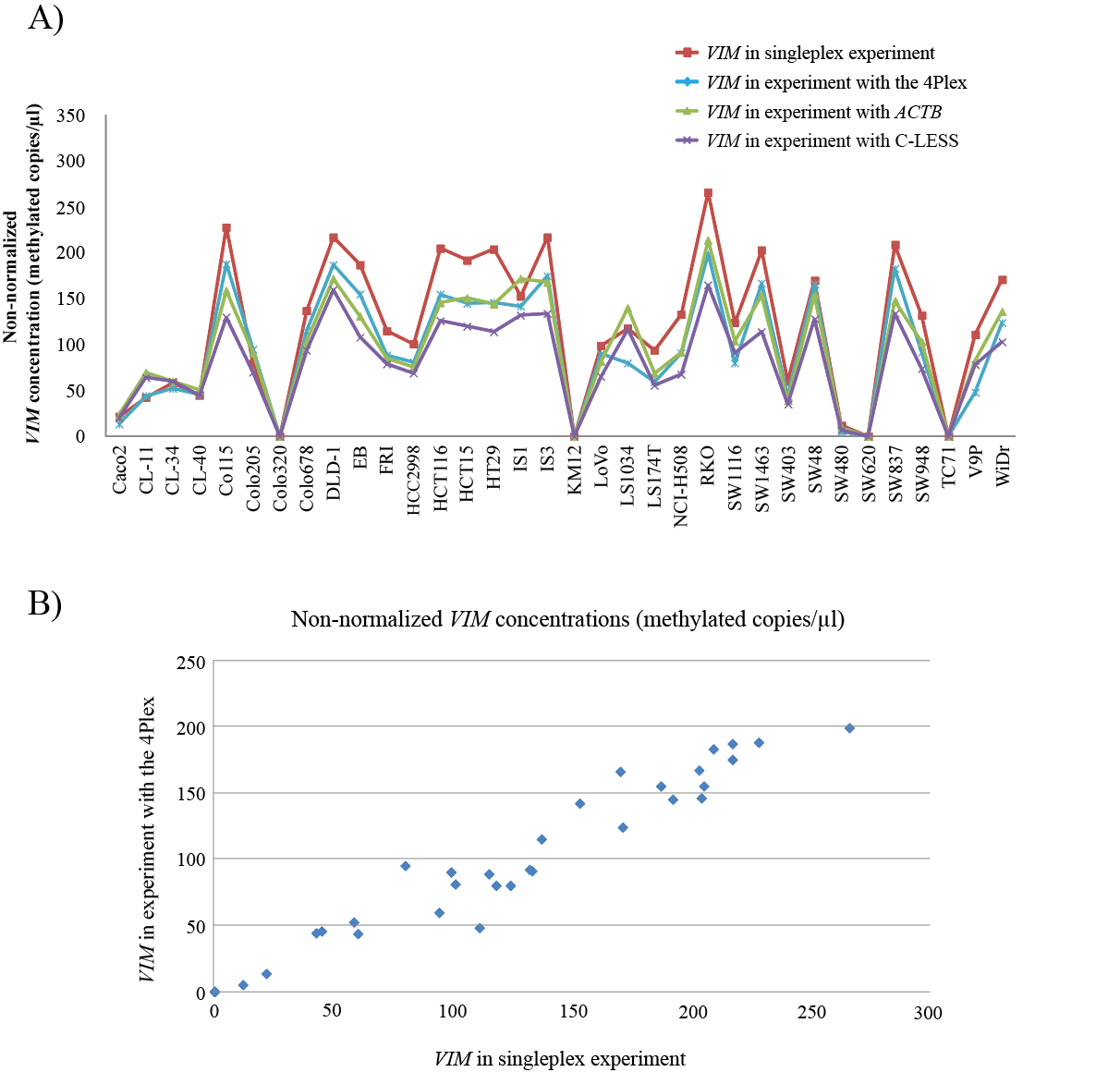


**Figure S4: Non-normalized *VIM* concentrations are lower with a control assay included in the reaction.** A) Non-normalized *VIM* concentrations are shown across the cell line panel for an experiement without an internal control (red), and from experiments including either of the three controls; 4Plex (blue), *ACTB* (green), and C-LESS (purple). B) Non-normalized conentrations in methylated copies/ul is plotted for *VIM* in a singleplex experiment (x-axis) against *VIM* in combination with the 4Plex (y-axis).


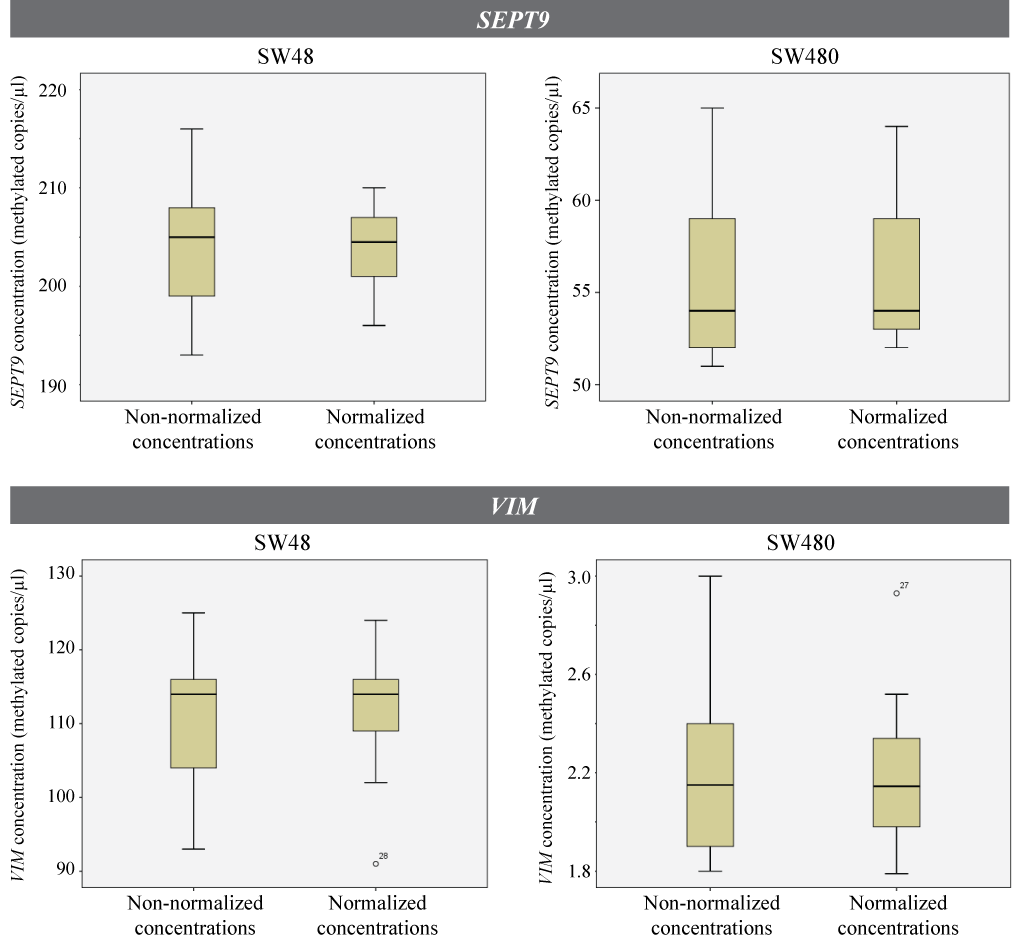


**Figure S5: A tendency of lower variation in 4Plex-normalized target gene concentrations is seen in replicates of the same sample.** Non-normalized and 4Plex-normalized concentrations of *SEPT9* (upper panel) and *VIM* (lower panel) are shown for replicates of two different samples (SW48 and SW480). Each analysis includes 14 replicates.

**
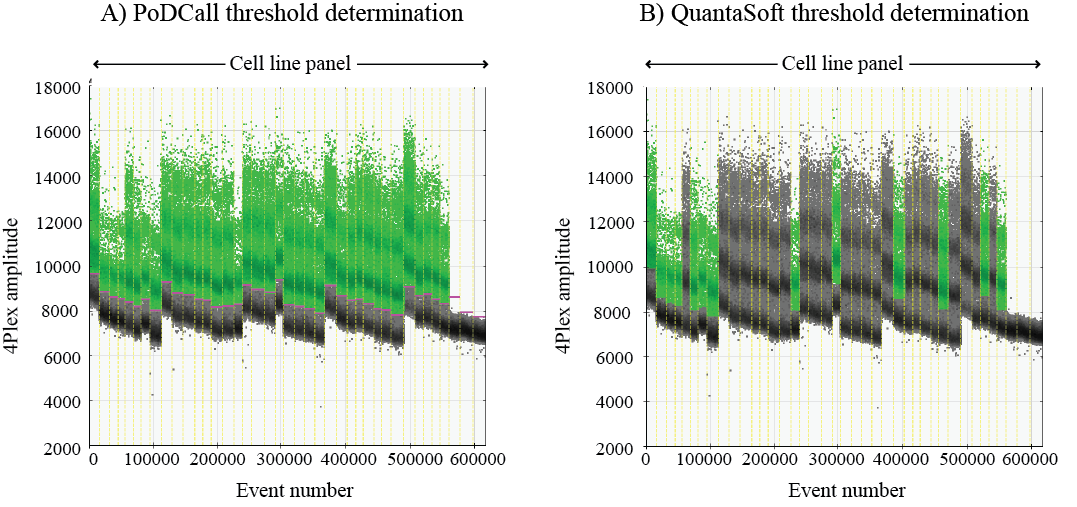
**

**Figure S6: PoDCall, the algorithm for automated threshold determination, corrects for shifts in baseline fluorescence between samples and performs better than the QuantaSoft software.** X-axis: the accumulative number of droplets for the analyzed wells. The yellow vertical dotted lines separate individual wells. Y-axis: fluorescence amplitude value of the 4Plex. Positive droplets are shown in green, and negative in black/grey. (A) Threshold determination by PoDCall has a 100% success rate (40/40 wells). Thresholds are represented as pink bars. (B) Threshold determination by the QuantaSoft software for the same experiment has a 40% success rate (16/40 wells).
