# Supplementary material for: A robust internal control for high-precision DNA methylation analyses by droplet digital PCR
Source: Clin Epigenetics. 2018 Feb 21;10:24. doi: 10.1186/s13148-018-0456-5 (PMC5822558; doi:10.1186/s13148-018-0456-5)
Supplement: Supplementary file 2 — dMIQE checklist for authors, reviewers, and editors. (DOCX 262 kb) [file 13148_2018_456_MOESM2_ESM.docx]

Additional file 2

**dMIQE checklist for authors, reviewers, and editors**

| **Item to check** | **Importance** | **Comment** |
| --- | --- | --- |
| **Experimental design** |  |  |
| Definition of experimental and control groups. | E | Not relevant |
| Number within each group. | E | Not relevant |
| Assay carried out by core lab or investigator's lab? | D | Not relevant |
| Power analysis. | D | Not relevant |
| **Sample** |  |  |
| Description. | E | DNA extracted from cell lines |
| Volume or mass of sample processed. | E | Not relevant |
| Microdissection or macrodissection. | E | Not relevant |
| Processing procedure. | E | Not relevant |
| If frozen—how and how quickly? | E | Snap-frozen at -80°C |
| If fixed—with what, how quickly? | E | Not relevant |
| Sample storage conditions and duration (especially for formalin-fixed, paraffin-embedded samples). | E | Stored at -80°C for up to 9 months prior to DNA isolation |
| **Nucleic acid extraction** |  |  |
| Quantification—instrument/method. | E | NanoDrop 1000 Spectrophotometer (Thermo Fisher Scientific) |
| Storage conditions: temperature, concentration, duration, buffer. | E | 4°C, ~100ng/µl, up to 4 years prior to bisulfite conversion, 1xTE-buffer |
| DNA or RNA quantification | E | DNA quantification |
| Quality/integrity, instrument/method, e.g. RNA integrity/R quality index and trace or 3′:5′. | E | Not relevant |
| Template structural information. | E | DNA stored without further modifications prior to bisulfite conversion |
| Template modification (digestion, sonication, preamplification, etc.). | E | Bisulfite conversion, see ”Materials and Methods” |
| Template treatment (initial heating or chemical denaturation). | E | Bisulfite conversion, see ”Materials and Methods” |
| Inhibition dilution or spike. | E | No |
| DNA contamination assessment of RNA sample. | E | Not relevant |
| Details of DNase treatment where performed. | E | Not performed |
| Manufacturer of reagents used and catalogue number | D | DNA extraction: Standard phenol-chloroform protocol or magnetic beads approach (Maxwell^®^ 16 System, Promega)  Bisulfite conversion: The EpiTect Bisulfite Kit (Qiagen) – Cat No./ID: 59104 |
| Storage of nucleic acid: temperature, concentration, duration, buffer. | E | 4°C, ~100ng/µl, up to 4 years prior to bisulfite conversion, 1xTE-buffer |
| **RT (If necessary)** |  |  |
| cDNA priming method + concentration. | E | Not relevant |
| One- or 2-step protocol. | E | Not relevant |
| Amount of RNA used per reaction. | E | Not relevant |
| Detailed reaction components and conditions. | E | Not relevant |
| RT efficiency. | D | Not relevant |
| Estimated copies measured with and without addition of RT.[^b^](http://clinchem.aaccjnls.org/content/59/6/892/tab-figures-data#fn-2) | D | Not relevant |
| Manufacturer of reagents used and catalogue number. | D | Not relevant |
| Reaction volume (for 2-step RT reaction). | D | Not relevant |
| Storage of cDNA: temperature, concentration, duration, buffer. | D | Not relevant |
| **dPCR target information** |  |  |
| Sequence accession number. | E | Additional file 1: Fig. S1 |
| Amplicon location. | D | Additional file 1: Fig. S1 |
| Amplicon length. | E | Additional file 1: Fig. S1 |
| In silico specificity screen (BLAST, etc.). | E | Not applicable, assays amplify a bisulfite converted version of the genome |
| Pseudogenes, retropseudogenes or other homologs? | D | - |
| Sequence alignment. | D | - |
| Secondary structure analysis of amplicon and GC content. | D | Checked in Methyl Express 3.0 (Applied Biosystems) |
| Location of each primer by exon or intron (if applicable). | E | All 4Plex assays are located in exons. More details available upon request. |
| Where appropriate, which splice variants are targeted? | E | Not relevant |
| **dPCR oligonucleotides** |  |  |
| Primer sequences and/or amplicon context sequence.[^b^](http://clinchem.aaccjnls.org/content/59/6/892/tab-figures-data#fn-2) | E | Additional file 1: Fig. S1 |
| RTPrimerDB (real-time PCR primer and probe database) identification number. | D | Not relevant |
| Probe sequences.[^b^](http://clinchem.aaccjnls.org/content/59/6/892/tab-figures-data#fn-2) | D | Additional file 1: Fig. S1 |
| Location and identity of any modifications. | E | Not relevant |
| Manufacturer of oligonucleotides. | D | Primers from BioNordika Bergman, probes from Life Technologies |
| Purification method. | D | RT-Cartridge |
| **dPCR protocol** |  |  |
| Complete reaction conditions. | E | See ”Materials and methods” |
| Reaction volume and amount of RNA/cDNA/DNA. | E | See ”Materials and methods” |
| Primer, (probe), Mg++ and dNTP concentrations. | E | See ”Materials and methods” |
| Polymerase identity and concentration. | E | The polymerase is included in the ‘2x ddPCR Supermix for Probes - No dUTP’ (BioRad) |
| Buffer/kit catalogue no. and manufacturer. | E | All reagents, instruments and equipments used are those recommended from the manufacturer (BioRad). |
| Exact chemical constitution of the buffer. | D | Not provided from manufacturer |
| Additives (SYBR green I, DMSO, etc.). | E | Not relevant |
| Plates/tubes Catalogue No and manufacturer. | D | - |
| Complete thermocycling parameters. | E | Additional file 1: Table S2 |
| Reaction setup. | D | See ”Materials and methods” |
| Gravimetric or volumetric dilutions (manual/robotic). | D | Volumetric dilutions (manual) |
| Total PCR reaction volume prepared. | D | 22 µl |
| Partition number. | E | Total partition number per well ≈ 15.000 droplets, Standard Deviation ≈ 2300 droplets (average across all experiments in the study) |
| Individual partition volume. | E | Average: 0.834 nL (inherent of the Bio-Rad Droplet Generator; Corbisier et al., Anal Bioanal Chem. 2015) |
| Total volume of the partitions measured (effective reaction size). | E | ~12.510 nL (~15.000droplets*0.834nL) |
| Partition volume variance/SD. | D | - |
| Comprehensive details and appropriate use of controls. | E | The methylation-positive control (*in vitro* methylated DNA) was amplifying as expected in all analyses (see figures below)  The methylation-negative control was found unmethylated in all analyses (normalized concentration of 0-0.1 copies/μl; see figures below)  The no-template control was negative in all analyses (normalized concentration of 0 copies/μl; see figures below)  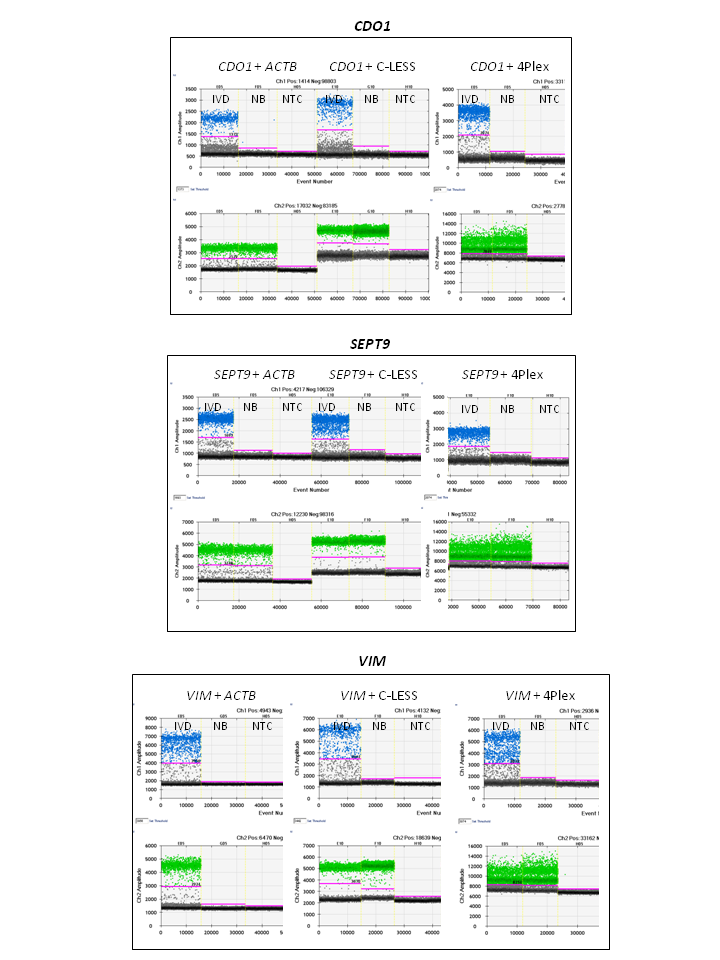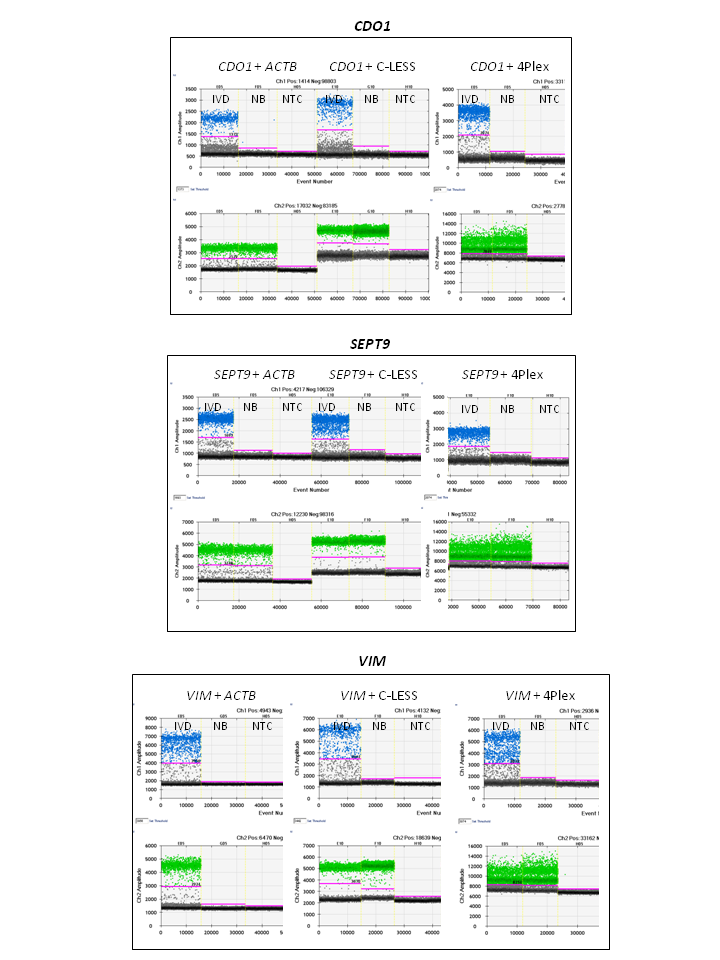  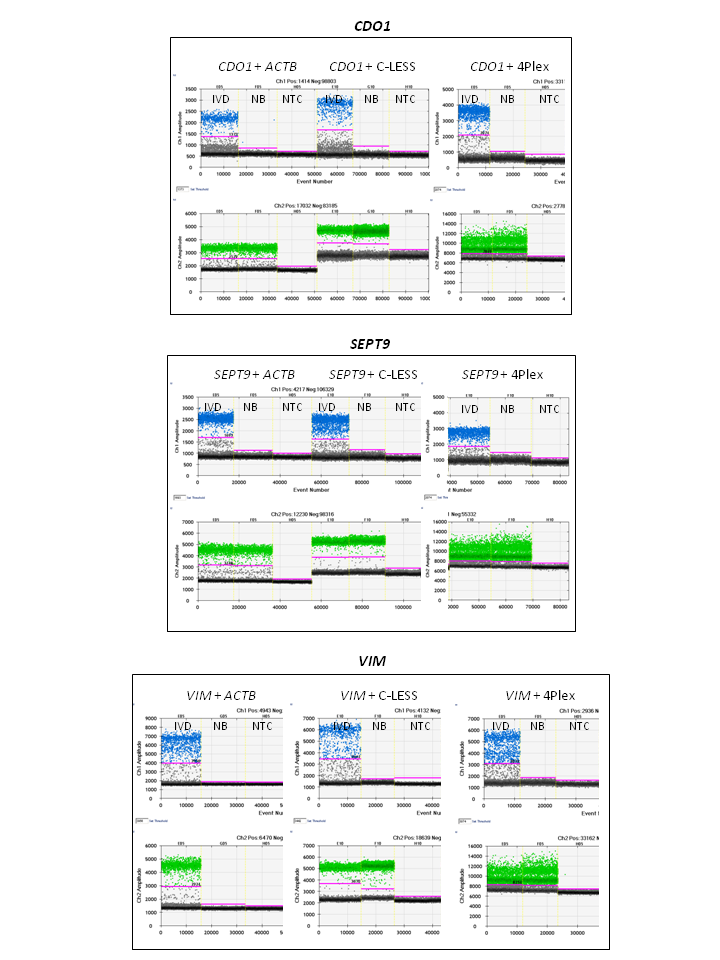 |
| Manufacturer of dPCR instrument. | E | BioRad |
| **dPCR validation** |  |  |
| Optimization data for the assay. | D | Assay have been optimized on the qMSP platform, information available upon request |
| Specificity (when measuring rare mutations, pathogen sequences etc.). | E | Not relevant |
| Limit of detection of calibration control. | D | - |
| If multiplexing, comparison with singleplex assays. | E | Yes, see Fig. 3 and Additional file 1: Fig. S4 |
| **Data analysis** |  |  |
| Mean copies per partition (λ or equivalent). | E | λ= –ln(1–*k/n*), where *k* = number of positive partitions, and *n* = number of partitions  Average λ: 0.16 (*CDO1*), 0.13 (*SEPT9*), 0.07 (*VIM*), 0.78 (4Plex), 0.17 (*ACTB*), 0.26 (C-LESS). |
| dPCR analysis program (source, version). | E | QuantaSoft version 1.7.4.0917 (BioRad) |
| Outlier identification and disposition. | E | By PoDCall, the in-house developed algorithm |
| Results of no-template controls. | E | Negative |
| Examples of positive(s) and negative experimental results as supplemental data. | E | See Fig. 5 for representative examples |
| Where appropriate, justification of number and choice of reference genes. | E | 4Plex as normalization reference |
| Where appropriate, description of normalization method. | E | See ”Materials and methods” |
| Number and concordance of biological replicates. | D | - |
| Number and stage (RT or dPCR) of technical replicates. | E | 12 technical replicates was run on dPCR for two representative samples (Fig. 6 and Additional file 1: Fig. S5). |
| Repeatability (intraassay variation). | E | Low intraassay variation (median difference for a representative sample≈4cop/µl) |
| Reproducibility (interassay/user/lab etc. variation). | D | - |
| Experimental variance or CI.[^d^](http://clinchem.aaccjnls.org/content/59/6/892/tab-figures-data#fn-2) | E | Average size of 95% Poisson error CI (cop/μl): *CDO1*~16, *SEPT9*~13, *VIM*~10, 4Plex ~ 43, *ACTB*~16, C-LESS~21. |
| Statistical methods used for analysis. | E | See ”Materials and Methods” |
| Data submission using RDML (Real-time PCR Data Markup Language). | D | Not relevant |

- All essential information (E) must be submitted with the manuscript. Desirable information (D) should be submitted if possible.
- ^b^Disclosure of the primer and probe sequence is highly desirable and strongly encouraged. However, since not all commercial predesigned assay vendors provide this information, when it is not available assay context sequences must be submitted [Bustin et al. (48)].
- ^c^Assessing the absence of DNA using a no-RT assay (or where RT has been inactivated) is essential when first extracting RNA. Once the sample has been validated as DNA free, inclusion of a no-RT control is desirable, but no longer essential.
- ^d^When single dPCR experiments are performed, the variation due to counting error alone should be calculated from the binomial (or suitable equivalent) distribution.
